# Complete Genomic Sequences of Highly Pathogenic H5N1 Avian Influenza Viruses Obtained Directly from Human Autopsy Specimens

**DOI:** 10.1128/MRA.01498-18

**Published:** 2018-12-06

**Authors:** Kantima Sangsiriwut, Mongkol Uiprasertkul, Sunchai Payungporn, Prasert Auewarakul, Kumnuan Ungchusak, Wasan Chantratita, Pilaipan Puthavathana

**Affiliations:** a Department of Preventive and Social Medicine, Faculty of Medicine Siriraj Hospital, Mahidol University, Bangkok, Thailand; b Department of Pathology, Faculty of Medicine Siriraj Hospital, Mahidol University, Bangkok, Thailand; c Department of Biochemistry, Faculty of Medicine, Chulalongkorn University, Bangkok, Thailand; d Department of Microbiology, Faculty of Medicine Siriraj Hospital, Mahidol University, Bangkok, Thailand; e Bureau of Epidemiology, Ministry of Public Health, Nonthaburi, Thailand; f Department of Pathology, Faculty of Medicine, Ramathibodi Hospital, Bangkok, Thailand; g Center for Research and Innovation, Faculty of Medical Technology, Mahidol University, Nakhon Pathom, Thailand; Georgia Institute of Technology

## Abstract

The complete genomic sequences of H5N1 highly pathogenic avian influenza (HPAI) viruses were directly obtained from lung, trachea, and colon tissues and an intestinal fecal sample of a patient by using the next-generation sequencing technique. This is the first report on complete H5N1 viral genomes from human autopsy specimens.

## ANNOUNCEMENT

The H5N1 highly pathogenic avian influenza (HPAI) virus belongs to the family Orthomyxoviridae. It is an enveloped virus with an eight-segmented, single-stranded RNA genome of negative polarity, with a length of about 13.5 kb in total. The eight segments contain the polymerase basic 1 (*PB1*), polymerase basic 2 (*PB2*), polymerase acidic (*PA*), hemagglutinin (*HA*), nucleoprotein (*NP*), neuraminidase (*NA*), matrix (*M*), and nonstructural (*NS*) genes (1). Previous investigators and our group reported the presence of H5N1 genomic RNA and antigenomic RNA in clinical specimens and various organs, including cerebrospinal fluid, throat swabs, lung, intestine, spleen, trachea, brain, heart, liver, kidneys, and lymph nodes (2–8). Nevertheless, none reported the presence of the entire genome of the H5N1 virus in autopsy tissues. To date, the H5N1 viral genome sequences deposited in the GenBank database belonged to the virus isolates.

Herein, we report the complete genome sequences of H5N1 HPAI viruses, directly obtained from lung, trachea, and colon tissues and an intestinal fecal sample of a patient who died of the disease in 2006. This study received approval from the Siriraj Hospital (Mahidol University) Institutional Review Board. Autopsy samples were collected by a pathologist using the standard technique for routine histologic analysis. These samples were stored frozen at −80°C until used. Total RNA was extracted from all specimens by using TRIzol reagent (Invitrogen, Carlsbad, CA) and was further subjected to cDNA synthesis by using the SuperScript III first-strand synthesis system (Invitrogen). The cDNA was used as the template for amplification of all 8 RNA segments with specific primers and Platinum Taq HiFi (Invitrogen). The *PB2*, *PB1*, *PA*, *HA*, *NP*, and *NA* genes were amplified with two pairs of specific primers and yielded PCR products with 100 to 200 bp overlapping. The *M* and *NS* genes were amplified as a single amplicon. The amplified products were purified using the QIAquick gel extraction kit (Qiagen, GmbH, Hilden, Germany). Full-genome sequencing was performed with 4 DNA library sets by using the GS Junior platform (Roche Diagnostics, Basel, Switzerland). Briefly, 500 ng of each amplified DNA was pooled, and then DNA libraries were prepared by using a GS DNA library preparation kit. The DNA libraries were subjected to emulsion PCR (emPCR) by using the GS emPCR kit I according to the manufacturer's instructions. After recovery and enrichment, the beads were sequenced using the GS Junior platform. The raw sequencing data (FASTQ) were trimmed based on the quality score. Low-quality data (Q score, <30) and adaptor sequences were removed from the sequencing reads. The passed filter (PF) reads (Q score, >30) were used for mapping with the A/Thailand/1(KAN-1)/2004 reference genome (GenBank accession numbers AY626144 to AY626149, AY555150, and AY555151) (9) by using the map reads to reference command with default parameters of the CLC Genomics Workbench version 8.0.1. The read lengths, numbers of reads, and coverage levels are summarized in Table 1.

**TABLE 1:** H5N1 HPAI viral genome information by clinical specimen or organ

Parameter	Lung	Trachea	Colon	Feces
Avg read length (bp)	241.98	309.01	241.93	220.71
No. of total reads	116,464	165,494	210,112	123,005
No. of mapped reads	114,713	163,740	206,998	120,715
% of mapped reads	98.50	98.94	98.52	98.14
Coverage level				
*PB2*	1,921.38	3,548.50	3,539.18	1,923.36
*PB1*	2,314.26	4,231.17	4,019.37	2,581.20
*PA*	2,131.53	3,562.35	3,818.51	2,192.57
*HA*	466.53	1,398.66	1,796.84	1,549.18
*NP*	1,503.16	3,317.79	3,151.90	1,615.38
*NA*	2,816.06	3,820.49	4,009.57	897.39
*M*	3,154.45	5,908.40	5,804.05	3,269.52
*NS*	3,348.56	6,310.87	5,400.61	1,928.98
Avg	2,206.99	4,012.28	3,942.50	1,994.70

Our analyses on the full genomic sequences derived from the four clinical specimens demonstrated five nonsynonymous substitutions. The substitutions M315I in *PB2*, M628V in *PA*, and K150R and S202N in *NP* were present in the lung, colon, and intestinal feces, while V255F in *NA* was present in the trachea, colon, and intestinal feces.

### Data availability.

The viral sequences were deposited in GenBank under the accession numbers MG668904 to MG668911 for colon, MG668920 to MG668927 for trachea, MG668928 to MG668935 for lung, and MG668912 to MG668919 for the fecal specimen. The raw reads were deposited in the Sequence Read Archive under the BioProject accession number PRJNA494792.
